# Cannabidiol Increases Seizure Resistance and Improves Behavior in an *Scn8a* Mouse Model

**DOI:** 10.3389/fphar.2022.815950

**Published:** 2022-01-26

**Authors:** Lindsey Shapiro, Andrew Escayg, Jennifer C. Wong

**Affiliations:** Department of Human Genetics, Emory University, Atlanta, GA, United States

**Keywords:** *SCN8A*, sodium channel, epilepsy, seizure, behavior, mouse

## Abstract

Voltage-gated sodium channel genes are an important family of human epilepsy genes. *De novo* missense mutations in *SCN8A* (encoding Na_v_1.6) are associated with a spectrum of clinical presentation, including multiple seizure types, movement disorders, intellectual disability, and behavioral abnormalities such as autism. Patients with *SCN8A* mutations are often treated with multiple antiepileptic drugs, the most common being sodium channel blockers. Cannabidiol (CBD) has been included as a component of treatment regimens for some *SCN8A* patients; however, to date, there are no clinical trials that have evaluated the therapeutic potential of CBD in patients with *SCN8A* mutations. In the current manuscript, we demonstrated a dose-dependent increase in seizure resistance following CBD treatment in mice expressing the human *SCN8A* mutation R1620L (RL/+). We also found that CBD treatment improved social behavior and reduced hyperactivity in the RL/+ mutants. Our findings suggest that CBD may be beneficial in patients with *SCN8A-*associated disease.

## Introduction

Cannabidiol (CBD), the predominant non-psychomimetic constituent of cannabis, was recently approved for the treatment of several forms of severe pediatric epilepsy, and there are currently ongoing clinical trials for the use of CBD in autism (Pretzsch et al., 2019; Aran et al., 2021) and schizophrenia (Leweke et al., 2012; Mcguire et al., 2018). In rodent models, CBD has been shown to have anxiolytic (Campos and Guimaraes 2008; Schiavon et al., 2016), anti-depressive (Reus et al., 2011; Sales et al., 2020), pro-social (Cheng et al., 2014; Kaplan et al., 2017), and anti-inflammatory effects (Ruiz-Valdepenas et al., 2011; Mecha et al., 2013). There is also evidence that CBD can promote neurogenesis in the dentate gyrus (Schiavon et al., 2016).

Epilepsy affects 0.5–1% of the population and is characterized by recurring seizures that often manifest during childhood. Voltage-gated sodium channel (VGSC) genes are an important family of human epilepsy genes. *De novo* loss-of-function mutations in the VGSC *SCN1A* (encoding Na_v_1.1) are the main cause of Dravet syndrome (DS), a catastrophic early-life encephalopathy associated with prolonged and recurrent early-life febrile seizures, refractory afebrile epilepsy, and behavioral deficits (Claes et al., 2001; Claes et al., 2009; Lossin 2009; Escayg and Goldin 2010). CBD was recently approved by the FDA for use in three severe pediatric epilepsies, including DS where it was shown to significantly reduce spontaneous seizure frequency (Devinsky et al., 2016; Devinsky et al., 2017; Devinsky et al., 2019).

The first *SCN8A* mutation in a patient with epilepsy was identified in 2012 (Veeramah et al., 2012), and as such, treatments for patients with *SCN8A* mutations are not yet as well-defined as for other sodium channelopathies like DS. *De novo* missense mutations in *SCN8A* (encoding Na_v_1.6) are associated with a spectrum of clinical presentation, including multiple seizure types, movement disorders, intellectual disability, and behavioral abnormalities such as autism (Larsen et al., 2015; Butler et al., 2017; Gardella et al., 2018). There are currently no specific treatments for *SCN8A*-associated disease. Patients with *SCN8A* mutations are often treated with multiple antiepileptic drugs (AEDs), the most common being sodium channel blockers, like oxcarbazepine (Gardella et al., 2018; Schreiber et al., 2020). Mutations in *SCN8A* can result in increased neuronal excitability, in part, by increasing persistent and/or resurgent sodium currents (Pan and Cummins 2019; Wengert et al., 2019; Tidball et al., 2020). *In vitro* studies have demonstrated that CBD can reduce resurgent and persistent sodium currents (Patel et al., 2016; Ghovanloo et al., 2018). While CBD is included in treatment regimens for some *SCN8A* patients (Gardella et al., 2018; Trivisano et al., 2019; Schreiber et al., 2020), clinical trials to evaluate the therapeutic potential of CBD in patients with *SCN8A* mutations have not yet been conducted. Previous studies have evaluated the effect of CBD on induced seizures in wild-type rodent models (Klein et al., 2017; Vilela et al., 2017; Patra et al., 2019) and more recently, in mouse models of DS (Kaplan et al., 2017; Anderson et al., 2020). In the current manuscript, we provide the first evaluation of CBD in a mouse model of *Scn8a-*associated epilepsy. We demonstrate that CBD significantly increases resistance to induced seizures, improves social behavior, and reduces hyperactivity in this model.

## Materials and Methods

### Animals

Male heterozygous *Scn8a* ^R1620L/+^ (RL/+) mutants were bred with female C57BL/6J mice (Strain: 000,664, Jackson Laboratories) to generate RL/+ and wild-type (WT) offspring; genotyping was performed as previously described (Wong et al., 2021b). All animals were 3–4 months of age at the time of seizure and behavior testing. Mice were housed on a 12-h light/dark cycle with food and water *ad libitum*. Experiments were performed in accordance with the guidelines of the Institutional Animal Care and Use Committee of Emory University.

### Pharmaceutical Compounds

Cannabidiol (CBD, Cayman Chemical) was dissolved in a 1:1:18 ratio of 100% ethanol, cremophore, and 0.9% saline, respectively as previously described (Klein et al., 2017). CBD or vehicle was administered (intraperitoneal, i. p.) 2 hours prior to seizure induction or behavioral assessments. Pentylenetetrazole (PTZ, Sigma-Aldrich) was dissolved in 0.9% saline.

### Seizure Induction

#### 6 Hz

6 Hz seizures were induced as previously described (Barton et al., 2001; Devinsky et al., 2016; Wong et al., 2016; Lamar et al., 2017; Shapiro et al., 2019; Wong et al., 2019; Wong et al., 2021a). RL/+ and WT mice were subjected to a brief corneal stimulation (6 Hz, 0.2 ms pulse width, 3 s) using a constant current device (ECT unit, 57800; Ugo Basile, Comerio, Italy). Following electrical stimulation, mice were observed for behavioral seizures that were scored using a modified Racine scale (RS): RS0, no abnormal behavior; RS1, immobile ≥3 s; RS2, forelimb clonus, head bobbing, paw waving; and RS3, rearing and falling.

#### Pentylenetetrazole

PTZ seizures were induced as previously described (Shapiro et al., 2019; Wong et al., 2019; Wong et al., 2021a; Wong et al., 2021b). RL/+ and WT littermates were administered PTZ (100 mg/kg) subcutaneously, and latencies to the first myoclonic jerk (MJ) and generalized tonic-clonic seizure (GTCS) were recorded over a 30-min period.

### Behavioral Assessments

All behavioral assessments were analyzed by an experimenter blinded to genotype and treatment. Spontaneous seizures were not observed during any experiment. ANY-Maze behavior tracking software (Stoelting) was used to score social behavior and locomotor activity in the three-chamber social interaction paradigm and open field, respectively.

#### Three-Chamber Social Interaction

Sociability and social discrimination were assessed as previously described (Inglis et al., 2020; Wong et al., 2021a; Wong et al., 2021b). Mice were placed in the center chamber of a three-chamber apparatus. Each chamber (20 × 40 × 22 cm) was separated by a Plexiglas partition that had a small opening at the base (5 × 5 cm) to allow access to all of the chambers. A wire cup was used as the inanimate object or an enclosure for the stranger mice. Five pairs of age- and sex-matched C57BL/6J mice (Strain: 000664, Jackson laboratories) were used as “stranger mice.” These mice were acclimated to the wire cups within the three-chamber apparatus the day prior to experimental testing. Experimental mice were subjected to three consecutive trials. In Trial 1, an empty wire cup was placed in both the left and right chambers of the apparatus. The experimental mouse was placed into the center chamber and allowed to explore the apparatus for 10 min. In Trial 2, a stranger mouse was placed beneath one of the previously empty wire cups, and the experimental mouse was allowed to freely explore for 10 min. In Trial 3, a novel mouse was placed beneath the previously empty wire cup; therefore, the experimental mouse had the choice of exploring the now familiar mouse (from Trial 2) or the novel mouse. Sociability was defined as the time spent exploring the stranger mouse vs inanimate object (Trial 2). Social discrimination was defined as the time spent exploring the novel vs familiar mouse (Trial 3).

#### Open Field

Locomotor activity and anxiety were assessed as previously described (Inglis et al., 2020; Wong et al., 2021a; Wong et al., 2021b). Mice were placed in an apparatus (60 × 60 × 60 cm) and allowed to freely explore for 10 min. The distance traveled, average speed, and time spent in the center of the apparatus were recorded.

#### Rotarod

Motor coordination was assessed as previously described (Wong et al., 2021b; Shapiro et al., 2021). Mice were given two 1-minute practice trials on a fixed speed rotarod (5 RPM, Columbus Instruments, Columbus, OH). After the two practice trials, mice were tested on an accelerating rotarod (0–40 RPM) for up to 5 min. The latency to fall was recorded.

### qRT-PCR

Whole brains were extracted from P20-P21 WT and RL/+ mice. RNA extraction and cDNA synthesis were performed as previously described (Lamar et al., 2017; Wong et al., 2021b). PCR amplification of 5HT_1A_, 5HT_2A_, CB1R, CB2R, TRPV1, and GPR55 were performed. Table 1 provides the primer pairs used for PCR amplification. Analyses were conducted in technical triplicates using the Real-Time PCR Detection System and SYBR Green (BioRad). Expression levels were normalized to beta-actin.

**TABLE 1:** Primers used for qRT-PCR analyses of target CBD receptors.

Target	Primer pair
*5HT*_*1A*_	F: GAC​AGG​CGG​CAA​CGA​TAC​T
	R: CCA​AGG​AGC​CGA​TGA​GAT​AGT​T
*5HT*_*2A*_	F: TGG​ATG​TGC​TCT​TCT​CCA​CG
	R: TGG​CAT​GGA​TAT​ACC​TAC​GGA
*TRPV1*	F: AGG​GAG​ATC​CAC​GAA​CCA​GA
	R: GTT​GGG​GGT​CTC​ACT​GCT​AC
*CB1R*	F: GTG​TTC​CAC​CGC​AAA​GAT​AGT
	R: GCC​TGT​GAA​TGG​ATA​TGT​ACC​TG
*CB2R*	F: CAT​CTG​CGA​AAG​TGT​GAG​AGC
	R: GTC​CCA​GAA​GAC​TGG​GTG​TCA
*GPR55*	F: TCA​AGG​CTG​GGA​CTC​ATT​GG
	R: GCT​GCA​AGG​TTC​TGG​TAA​GC
*Beta-actin*	F: CAG​CTT​CTT​TGC​AGC​TCC​TT
	R: ACG​ATG​GAG​GGG​AAT​ACA​GC

### Statistical Analyses

All data are presented as mean ± SEM with *p* ≤ 0.05 considered statistically significant. All statistical analyses were performed with Prism 9.0 (GraphPad software, San Diego, CA). A Kruskal-Wallis test followed by Dunn’s multiple comparisons was used to compare Racine scores following 6 Hz induction. A log-rank Mantel Cox test was used to compare curves following PTZ administration. A two-way ANOVA followed by Sidak’s multiple comparisons test was used to compare behavioral assessments between genotypes (RL/+ or WT) and treatments (CBD or vehicle). A Mann-Whitney test was used to compare gene expression levels between RL/+ mutants and WT littermates.

## Results

### Cannabidiol Increases Resistance to Induced Seizures in a Dose-Dependent Manner

**FIGURE 1:**
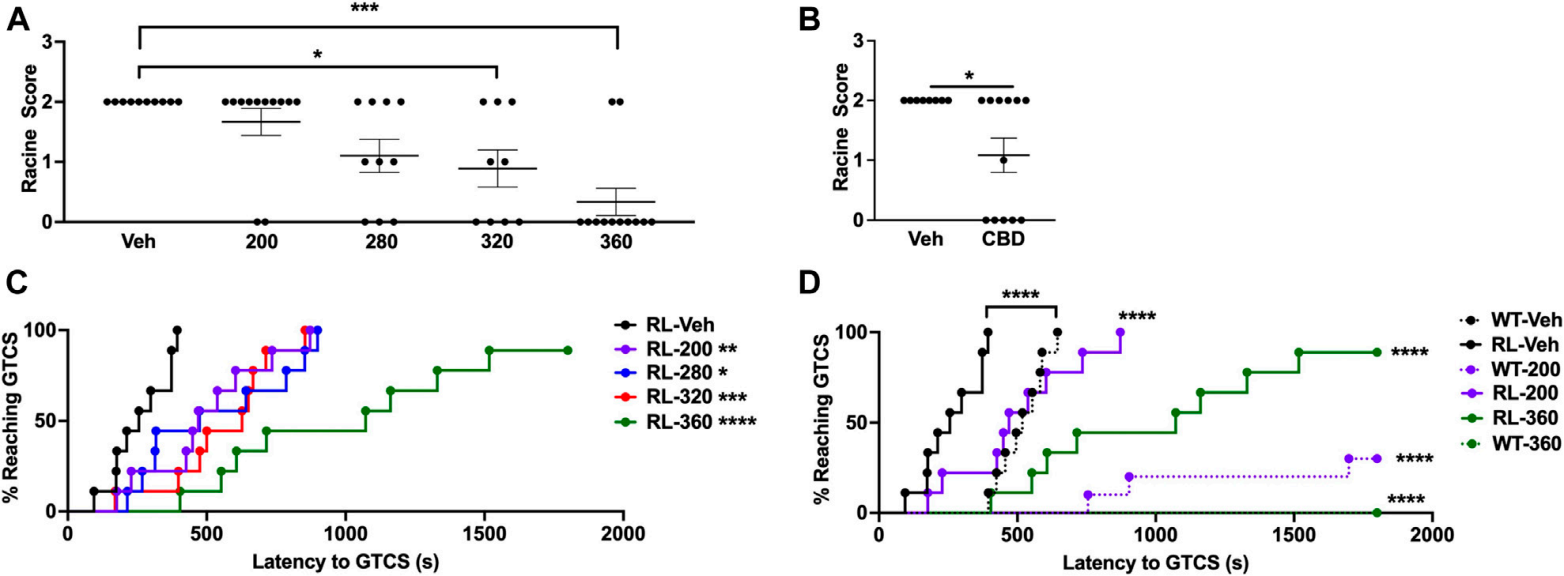
CBD significantly increases resistance against induced seizures. **(A)** A dose-response curve was generated using the 6 Hz seizure induction paradigm. Both 320 and 360 mg/kg CBD were able to protect against 6 Hz seizures in RL/+ mutants. *N* = 9–12/group. **(B)** We retested the highest dose of CBD (360 mg/kg) in a separate cohort of RL/+ mutants and found that it was also able to significantly increase resistance against 6 Hz seizures at twice the convulsive current (2xCC, 32 mA). *N* = 8–12/group. **(C)** We generated a dose-response curve using the PTZ seizure induction paradigm, and similarly observed a dose-dependent increase in the latency to the first generalized tonic-clonic seizure (GTCS). *N* = 9/group. Significance was determined when compared to vehicle-treated RL/+ mutants. **(D)** RL/+ mutants and WT littermates were treated with CBD (200 or 360 mg/kg) or vehicle and subjected to PTZ administration. We observed significant protection against PTZ-induced seizures with both doses of CBD. *N* = 8–10/group. Significance was determined when compared to vehicle-treated mice of the same genotype. *p ≤ 0.05, **p ≤ 0.01, ***p ≤ 0.001, ****p ≤ 0.0001.

We first generated a dose-response curve using the 6 Hz seizure induction paradigm following CBD administration. RL/+ mutants were administered CBD (200–360 mg/kg) or vehicle 2 hours prior to 6 Hz seizure induction (16 mA). As expected, all vehicle-treated RL/+ mutants exhibited a seizure (10 RS2; Figure 1A). We found that 320 and 360 mg/kg CBD were able to significantly increase resistance to 6 Hz seizures in RL/+ mutants. With 320 mg/kg CBD, 4/9 RL/+ mutants (44%) did not exhibit a seizure (4 RS0, 2 RS1, 3 RS2), and 10/12 RL/+ mutants (83%) were completely protected against 6 Hz seizures with 360 mg/kg CBD. Since we observed the greatest protection with 360 mg/kg CBD, we evaluated whether this dose would also protect against 6 Hz seizures when tested at twice the convulsive current (2×CC, 32 mA), which is used as a predictor of drugs that might protect against refractory seizures (Barton et al., 2001). At 2xCC, we found that 5 of 12 RL/+ mutants (42%) were completely protected against 6 Hz seizures when administered 360 mg/kg CBD (Figure 1B).

We similarly generated a dose-response curve using PTZ seizure induction. RL/+ mutants were administered CBD (200–360 mg/kg) or vehicle 2 h prior to PTZ administration (100 mg/kg). Each dose of CBD significantly increased the latency to the first PTZ-induced GTCS (Figure 1C), with the greatest increase in seizure latency observed with 360 mg/kg CBD. WT littermates were included for comparison at the lowest (200 mg/kg) and highest (360 mg/kg) doses of CBD tested (Figure 1D). Significant increases in the latency to the first PTZ-induced GTCS were observed with both doses in the WT littermates and RL/+ mutants; however, relatively greater protection was observed in the WT littermates as indicated by the larger rightward shift in the GTCS latency curves (Figure 1D). Furthermore, in the WT littermates, 7/10 and 8/8 mice that received 200 and 360 mg/kg CBD, respectively, did not exhibit a GTCS. While CBD significantly increased the latency to the PTZ-induced GTCS in the RL/+ mutants, it failed to block GTCS generation. All 9 RL/+ mutants exhibited a GTCS following treatment with 200 mg/kg CBD, and only 1 RL/+ mutant (1/9) administered 360 mg/kg CBD did not exhibit a GTCS (Figure 1D).

### Cannabidiol Significantly Improves Social Discrimination in RL/+ Mutants

**FIGURE 2:**
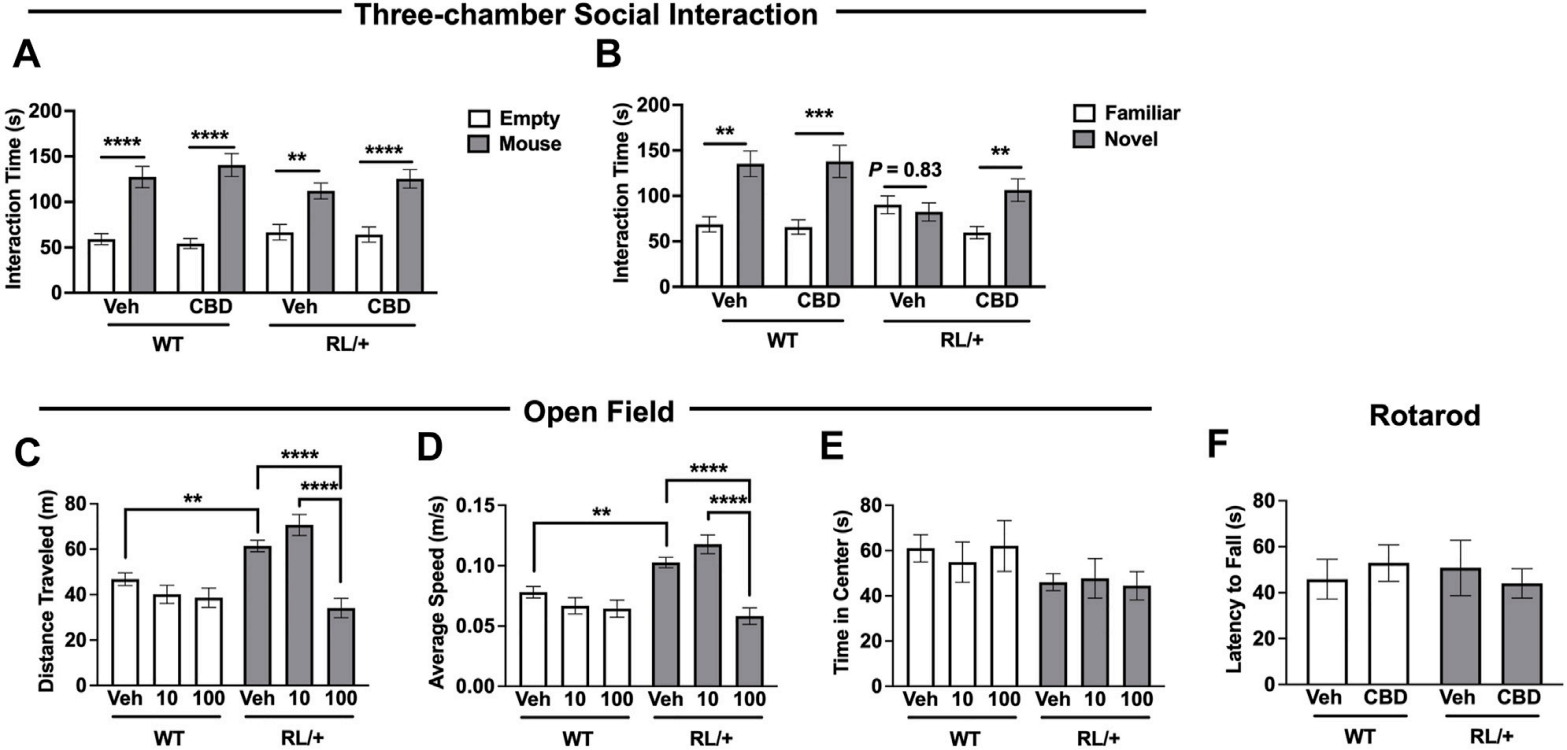
Cannabidiol improves social behavior and reduces hyperactivity in RL/+ mutants. **(A)** RL/+ mutants and WT littermates displayed normal sociability following vehicle or CBD treatment. **(B)** Vehicle-treated RL/+ mutants exhibited deficits in social discrimination; however, CBD (10 mg/kg) was able to restore more normal social discrimination in the mutants. **(A,B)** *N* = 12-13/group. **(C,D)** Following the administration of CBD (100 mg/kg), distance traveled by the RL/+ mutants **(C)** and their average speed **(D)** were comparable to WT littermates. CBD (10 mg/kg) had no effect on locomotor activity regardless of genotype. **(E)** The time spent in the center of the apparatus was comparable across both genotypes and treatment groups. **(C–E)** *N* = 12**–**26/group. **(F)** CBD (100 mg/kg) does not impair motor coordination in RL/+ mutants and WT littermates. *N* = 8**–**9/group. **p ≤ 0.01, ***p ≤ 0.001, ****p ≤ 0.0001.

Kaplan and others previously demonstrated that CBD (10–100 mg/kg) was able to ameliorate some behavioral abnormalities in a mouse model of DS (Kaplan et al., 2017). Therefore, we evaluated whether CBD could also improve behavior in the RL/+ mutants. We recently reported that RL/+ mutants have deficits in social discrimination (Wong et al., 2021a); therefore, we explored the ability of CBD to ameliorate this deficit. RL/+ mutants and WT littermates were administered 10 mg/kg CBD and social behavior was evaluated using the three-chamber social interaction paradigm. As expected, vehicle-treated WT littermates displayed normal sociability (Figure 2A) and social discrimination (Figure 2B), and importantly, CBD administration did not alter social behavior in these mice (Figure 2). Consistent with our previous report, vehicle-treated RL/+ mutants displayed normal sociability (Figure 2A) but failed to show the expected preference for novel versus familiar mice, demonstrating a deficit in social discrimination (Figure 2B). However, following CBD treatment, RL/+ mutants spent significantly more time interacting with the novel mice (Figure 2B), suggesting that CBD is able to restore more normal social discrimination in the RL/+ mutants.

### Cannabidiol Reduces Hyperactivity in RL/+ Mutants

To evaluate locomotor activity, RL/+ mutants and WT littermates were administered CBD (10 or 100 mg/kg) or vehicle and placed into an open field apparatus 2 hours later. Consistent with our previous analysis (Wong et al., 2021a), vehicle-treated RL/+ mutants were hyperactive as evidenced by traveling farther and faster compared to vehicle-treated WT littermates (Figures 2C,D). Locomotor activity in the WT littermates was not affected by either dose of CBD. Locomotor activity in the RL/+ mutants was not altered by 10 mg/kg CBD. However, RL/+ mutants that received 100 mg/kg CBD traveled significantly less and at a slower speed compared to vehicle-treated RL/+ mutants and were comparable to vehicle-treated WT littermates (Figures 2C,D), demonstrating that 100 mg/kg CBD was able to reduce hyperactivity in the RL/+ mutants. Regardless of genotype, we did not observe any effect of CBD on the time spent in the center of the open field apparatus (Figure 2E). We next evaluated whether 100 mg/kg CBD affected motor coordination using an accelerating rotarod. Motor coordination was comparable between RL/+ mutants and WT littermates administered 100 mg/kg CBD (Figure 2F), demonstrating that there are no motor toxicity effects with this dose of CBD.

### RL/+ Mutants and WT Littermates Have Comparable Expression Levels of CBD Target Receptors

**FIGURE 3:**
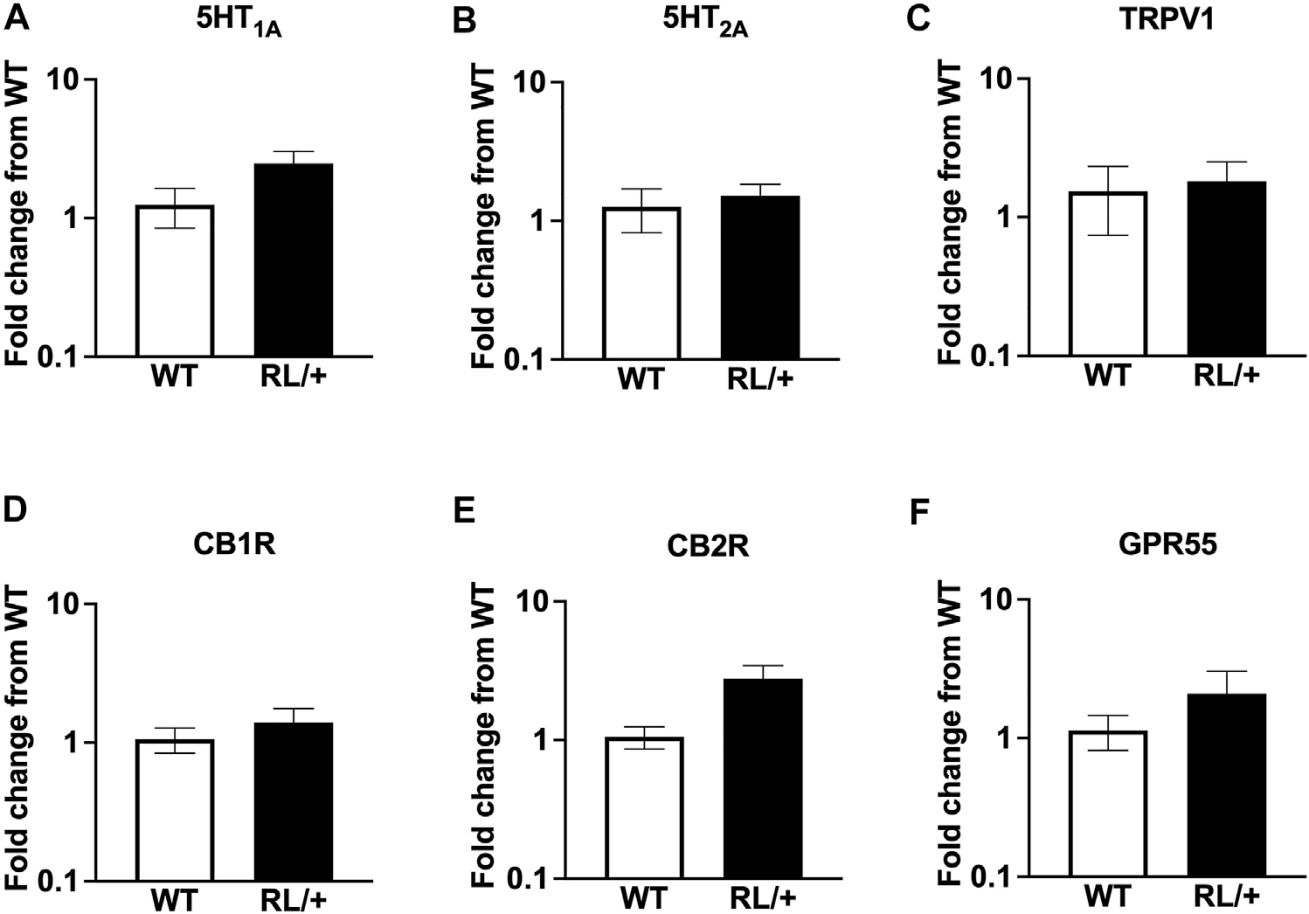
RL/+ mutants and WT littermates have comparable expression levels of target CBD receptors: **(A)** 5HT_1A_, **(B)** 5HT_2A_, **(C)** TRPV1, **(D)** CB1R, **(E)** CB2R, and **(F)** GPR55.

To determine whether RL/+ mutants and WT littermates differed in levels of mRNA of several known CBD target receptors, we performed qRT-PCR from whole brain tissue. We observed no statistically significant differences in expression of 5HT_1A_, 5HT_2A_, TRPV1, CB1R, CB2R, or GPR55 between RL/+ mutants and WT littermates (Figures 3A–F).

## Discussion

In the current study, we investigated the therapeutic potential of CBD in RL/+ mutant mice. To our knowledge, this is the first report evaluating CBD in an *Scn8a* mouse model. The *SCN8A* R1620L mutation was previously identified in a patient that presented with a wide range of behavioral abnormalities, including social behavior deficits, autism, attention deficit hyperactivity disorder, and behavioral seizures without accompanying electrographic activity (Rossi et al., 2017; Liu et al., 2019). Like the patient, RL/+ mutant mice exhibit a number of behavioral abnormalities, including deficits in social behavior, learning and memory, and the mice are hyperactive (Wong et al., 2021a). The RL/+ mutants also exhibit increased seizure susceptibility and infrequent spontaneous seizures (Wong et al., 2021a).

There is increasing interest in the use of CBD for the treatment of epilepsy, and accordingly, the therapeutic potential of CBD has been evaluated in a number of preclinical models. The Epilepsy Therapy Screening Program (ETSP) performed a systematic evaluation of the ability of CBD to increase resistance to induced seizures in WT CF1 mice and Sprague-Dawley rats (Klein et al., 2017; Patra et al., 2019). Those studies demonstrated that CBD was able to increase resistance to 6 Hz-induced seizures in mice and maximal electroshock induced-seizures in mice and rats (Klein et al., 2017).

In CF1 mice, the ETSP reported that 50 and 100% of the mice were protected against 6 Hz seizures at 2×CC with 164 and 200 mg/kg CBD, respectively (Klein et al., 2017). We found that higher CBD doses were required to achieve statistically significant protection against 6 Hz seizures in the RL/+ mutants (Figure 1A). At 2xCC, 5/12 RL/+ mutants (42%) were protected against 6 Hz seizures following administration of 360 mg/kg CBD (Figure 1B). While it is possible that greater protection might have been achieved with an even higher dose, we observed that RL/+ mutants exhibited a tremor, both when at rest and when active, following the administration of higher CBD doses. In addition, the TD_50_ for CBD (toxic dose, the dose at which 50% of the mice die) was previously reported to be approximately 425–500 mg/kg in WT mice (Klein et al., 2017). Therefore, higher CBD doses were not tested.

Interestingly, unlike the RL/+ mutants, no adverse effects were observed when their WT littermates were administered CBD. We also observed less seizure protection in the RL/+ mutants compared to their WT littermates. For example, while the latency to the first GTCS was significantly increased in the RL/+ mutants with 360 mg/kg CBD, 8/9 mutants still exhibited a GTCS. In contrast, none of the WT littermates exhibited a GTCS at this dose (Figure 1D).

Previous studies in mouse models of *Scn1a* dysfunction have demonstrated that CBD can reduce spontaneous seizure frequency, although some inconsistences have been observed. Kaplan et al. found that administration of CBD (100 mg/kg twice daily, i.p.) was able to reduce spontaneous seizure frequency in *Scn1a* ^*+/−*^ mutants during a period of increased seizure risk (P21-P28) (Kaplan et al., 2017). In contrast, Anderson et al. found no effect on spontaneous seizures when CBD (12 and 25 mg/kg) was administered to *Scn1a* ^*+/−*^ mutants in rodent chow (Anderson et al., 2020). Given the wide variability in occurrence and low frequency of spontaneous seizures in the RL/+ mutants (Wong et al., 2021a), we were unable to evaluate the effect of CBD on spontaneous seizures. In future studies, *Scn8a* mouse models that exhibit more frequent spontaneous seizures could be used to address this important question.

In addition to its potential therapeutic effects on seizure phenotypes, CBD has also been shown to improve some aspects of behavior (Campos and Guimaraes 2008; Reus et al., 2011; Schiavon et al., 2016; Sales et al., 2020). When administered to *Scn1a* ^*+/−*^ mutants, CBD (10–20 mg/kg) restored more normal social behavior and, at a higher dose (100 mg/kg), CBD also reduced locomotor activity to levels observed in WT littermates (Kaplan et al., 2017). Consistent with these observations, we found that similar doses of CBD were able to normalize social discrimination (Figure 2B) and locomotor activity (Figures 2C,D) in the RL/+ mutants. Interestingly, while high doses of CBD (320–360 mg/kg) were required to achieve seizure protection in the RL/+ mutants, much lower doses (10–100 mg/kg) provided robust improvement in behavior.

Given the different response to CBD between the RL/+ mutants and WT littermates, we speculated that the mutants may have different levels of expression of target CBD receptors (5HT_1A_, 5HT_2A_, TRPV1) (Rodrigues Da Silva et al., 2020) or receptors of the endocannabinoid system (CB1R, CB2R, GPR55) (Ryberg et al., 2007). However, mRNA levels of 5HT_1A_, 5HT_2A_, TRPV1, CB1R, CB2R, and GPR55 were found to be comparable between RL/+ mutants and WT littermates (Figures 3A–F). It is possible that differences between the RL/+ mutants and WT littermates in the expression of other CBD target receptors, CBD metabolism, or network excitability may have contributed to the altered response to CBD. Previous studies have identified several potential mechanisms by which CBD decreases neuronal excitability, including acting upon TRPV1 and blocking the orphan receptor GPR55 (Devinsky et al., 2014; Kaplan et al., 2017). At physiologically relevant concentrations, CBD does not directly bind to the endocannabinoid receptors CB1 or CB2 (Devinsky et al., 2014; Mcpartland et al., 2015). However, CBD has been shown to inhibit human and mouse Na_v_1.6 currents at therapeutically relevant concentrations (Ghovanloo et al., 2018). Furthermore, CBD has also been shown to reduce resurgent and persistent currents in both wild-type and HEK cells expressing the *SCN8A* N1768D mutation (Patel et al., 2016; Ghovanloo et al., 2018).

In the current manuscript, we provide the first evaluation of CBD in an *Scn8a* mouse model and demonstrated a dose-dependent increase in resistance against induced seizures. We also established that CBD can restore more normal social behavior and reduce hyperactivity in RL/+ mutants. Taking into consideration differences in the metabolic rate between mice and humans, the seizure protective dose in the RL/+ mutants (320–360 mg/kg) would correspond to an approximate human dose of 26–29 mg/kg (Nair and Jacob 2016). While an effective dose range for CBD in patients with *SCN8A* mutations has not yet been established, in a long-term open label trial in patients with DS and Lennox-Gastaut syndrome (GWPCARES, NCT02224573), CBD doses from 2.5 to 20 mg/kg/d were administered. In patients that did not gain seizure control, the dose of CBD was increased to 30 mg/kg/d (Devinsky et al., 2019; Thiele et al., 2019). Thus, the dose range of CBD used in the present study is consistent with current clinical application. Furthermore, our data raises the possibility that while higher doses may be necessary achieve seizure control, lower doses of CBD might ameliorate some behavioral deficits. Taken together, our findings suggest that CBD could represent a promising therapy for patients with *SCN8A* mutations.

## Data Availability

The original contributions presented in the study are included in the article, further inquiries can be directed to the corresponding author.
